# The green tea modulates large intestinal microbiome and exo/endogenous metabolome altered through chronic UVB-exposure

**DOI:** 10.1371/journal.pone.0187154

**Published:** 2017-11-08

**Authors:** Eun Sung Jung, Hye Min Park, Seung Min Hyun, Jong Cheol Shon, Digar Singh, Kwang-Hyeon Liu, Tae Woong Whon, Jin-Woo Bae, Jae Sung Hwang, Choong Hwan Lee

**Affiliations:** 1 Department of Bioscience and Biotechnology, Konkuk University, Seoul, Republic of Korea; 2 Department of Genetic Engineering & Graduate School of Biotechnology, Kyung Hee University, Yongin, Republic of Korea; 3 College of Pharmacy and Research Institute of Pharmaceutical Science, Kyungpook National University, Daegu, Republic of Korea; 4 Department of Life and Nanopharmaceutical Sciences and Department of Biology, Kyung Hee University, Seoul, Republic of Korea; Korea University, REPUBLIC OF KOREA

## Abstract

The attenuating effects of green tea supplements (GTS) against the ultraviolet (UV) radiation induced skin damages are distinguished. However, the concomitant effects of GTS on the large intestinal microbiomes and associated metabolomes are largely unclear. Herein, we performed an integrated microbiome-metabolome analysis to uncover the esoteric links between gut microbiome and exo/endogenous metabolome maneuvered in the large intestine of UVB-exposed mice subjected to dietary GTS. In UVB-exposed mice groups (UVB), class *Bacilli* and order *Bifidobacteriales* were observed as discriminant taxa with decreased lysophospholipid levels compared to the unexposed mice groups subjected to normal diet (NOR). Conversely, in GTS fed UVB-exposed mice (U+GTS), the gut-microbiome diversity was greatly enhanced with enrichment in the classes, *Clostridia* and *Erysipelotrichia*, as well as genera, *Allobaculum* and *Lachnoclostridium*. Additionally, the gut endogenous metabolomes changed with an increase in amino acids, fatty acids, lipids, and bile acids contents coupled with a decrease in nucleobases and carbohydrate levels. The altered metabolomes exhibited high correlations with GTS enriched intestinal microflora. Intriguingly, the various conjugates of green tea catechins *viz*., sulfated, glucuronided, and methylated ones including their exogenous derivatives were detected from large intestinal contents and liver samples. Hence, we conjecture that the metabolic conversions for the molecular components in GTS strongly influenced the gut micro-environment in UVB-exposed mice groups, ergo modulate their gut-microbiome as well as exo/endogenous metabolomes.

## Introduction

The gut microbiome actively mediates the pathogenesis in a myriad of metabolic disorders *viz*., obesity, diabetes, and inflammatory bowel diseases [1–4]. Hence, the importance of the gut microbiome and the evaluation of its health effects have progressively emerged as an important discipline. In general, the dietary nutrients influence the composition of gut microbial community through providing substrates for microbial metabolism [5]. In recent years, the compositions of gut microbiota and associated metabolomes have become a focus of research at the intersection of dietary elements and their effects on skin health. Previously, very few studies have highlighted the association between intestinal microbiome states and their correlations with cutaneous disorders. The profound correlations have been established between the intestinal microbial diversity and atopic eczema among infants [6]. Similarly, the oral administration of *Bifidobacterium breve* was reported to suppress the chronic UVB-induced changes related to mouse skin hydration and epidermal thickening [7]. However, the underlying mechanisms related to the effects of gut microflora on skin homeostasis has remained elusive.

The chronic UVB (280–315 nm) exposures harms skin tissues, concomitantly inducing the erythema and inflammation through enhancing the oxidative stress in vital organs and tissues viz., liver, skin, and blood [8, 9]. The global trends of average UVB exposure are varied with its higher levels at the equator and within tropics with seasonal spikes at the equinoxes [10]. However, the average UV exposure also depends on a variety of factors including the occupational, recreational, and geographical factors viz., sun elevation, latitude, altitude, cloud cover, ozone levels, and ground reflection [11]. Varieties of green tea components, particularly catechins, are potent anti-oxidants with associated anti-inflammatory activities, and hence mitigate a state of oxidative stress [12]. Previously, we have shown that the oral administration of green tea supplements attenuate UVB-induced alteration of skin metabolites in mice [13]. In case of green tea, the polyphenols such as epicatechin (EC), epigallocatechin (EGC), epicatechin gallate (ECG), and epigallocatechin gallate (EGCG) pivotally affects the gut microbial communities, and are subsequently bio-transformed into various bioactive components [14]. Thus, we hypothesize that green tea supplements (GTS) effectively ameliorates the UV induced skin damages through enriching the altered gut microbiomes and associated metabolic gamut maneuvered through a chain of metabolic conversions. However, we lack the substantiated literatures directly correlating the detrimental effects of UVB exposure on large intestine, especially, how the gut microbiome and metabolomes, mitigated through GTS. In this study, we aimed to establish a correlative microbiome-metabolome model underpinning the effects of chronic UVB irradiation on large intestinal microbiota of UVB treated mice groups with GTS alleviating the intestinal microbiome and exo/endogenous metabolomes.

## Material and methods

### Chemicals

Acetonitrile, methanol, and water were obtained from Fisher Scientific (Pittsburgh, PA, USA). Formic acid, pyridine, methoxyamine hydrochloride, *n*-methyl-*n*-(trimethylsilyl) trifluoroacetamide (MSTFA), and standard compounds were purchased from Sigma Chemical Co. (St. Louis, MO, USA).

### Animal experiments

**Fig 1:**
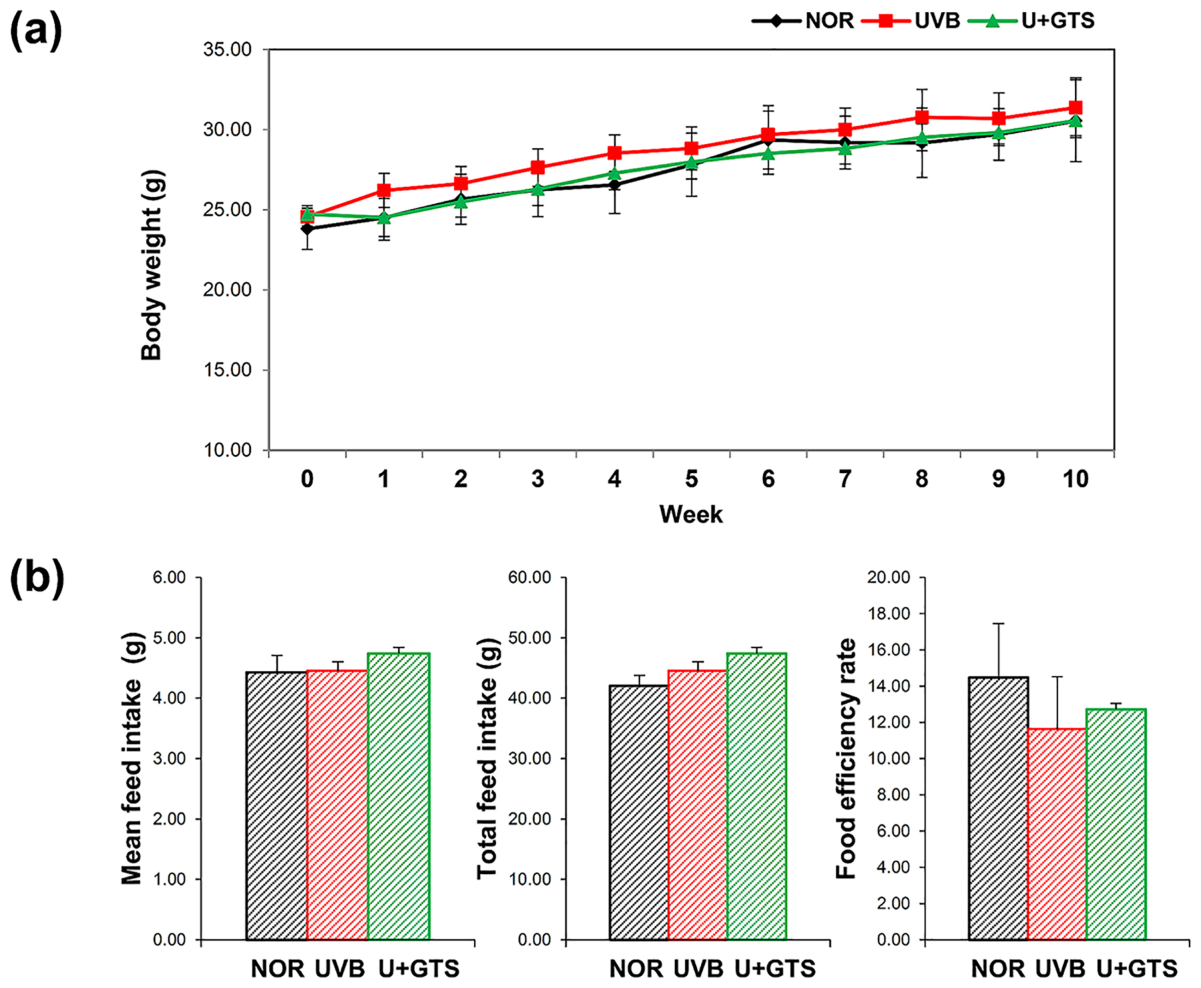
Histograms of a, body weight; b, food intake and food efficiency ratio of mice in each experimental groups. Body weight was measured every week. All the results were shown as mean ± standard deviation, and did not show significantly differences among groups. Black square-NOR group; red square-UVB group; green square- U+GTS group.

Female albino hairless mice (Skh:HR-1, 6–8 weeks old) were purchased from Orient Bio (Seongnam, Korea) and housed under controlled temperature (24 ± 2°C), humidity (55 ± 10%), and light (12 h light/dark cycle) conditions. After 1 week of acclimation, the mice were randomly divided into three groups: Control (NOR) (n = 12), UVB exposed (UVB) (n = 12), and UVB-exposed and fed on diet containing 1% green tea extract (U+GTS) (n = 12). Four fluorescent lamps (TL 20W/12 RS SLV, wavelength 290–390 nm, peak emission 315 nm; Philips, Amsterdam, Netherlands) were used for UVB irradiation, and monitored intensity with a UV meter (VARIOCONTROL, Waldmann ver. 2.03, Germany). UVB irradiation was initiated with 75 mJ/cm^2^ doses *i*.*e*.,1 minimal erythema dose or MED, ramped-up by 1 MED/week to 4 MED and maintained subsequently. The mice were exposed to UVB radiation three times a week. While UVB exposure, mice were moving freely in the cage, at a distance of 30 cm from the lamps. The NOR and UVB groups were fed a control diet (Ain-93G), whereas the U+GTS group was fed on 1% GTE diet (Ain-93G + 1% GTE). The GTE was provided by AmorePacific Corp. (Seoul, Korea), and contained 50% of the total catechins including EC, EGC, ECG, and EGCG. No significant differences in food intake and body weight of mice were observed among the groups based on weekly measurements (Fig 1). After 10 weeks of animal experiment, the mice were sacrificed and their liver as well as large intestine (including contents) were collected, and subsequently stored at deep freezing condition (−80°C). For preparing microbial DNA samples and metabolite extraction, we pulled out large intestinal contents from tissue. All the samples were used for metabolites extraction and four randomly selected samples were subjected to DNA preparation. All animal experimental procedures were approved by the Institutional Animal Care and Use Committee of Gyeonggi Institute of Science & Technology (permit number: 2013-12-0006), and were performed in accordance with their guidelines. The animal experimental procedures were done to minimize suffering.

### Intestinal microbial community analysis

#### DNA extraction and emulsion-based PCR (emPCR)

Extraction of bacterial DNA was performed using a stool PowerWater DNA Isolation Kit (MO BIO). A library was prepared using PCR products according to the GS FLX plus library prep guide. Libraries were quantified using the Picogreen assay (Victor 3). The emPCR, corresponding to clonal amplification of the purified library, was performed using the GS-FLX plus emPCR Kit (454 Life Sciences). Briefly, libraries were immobilized onto DNA capture beads. The library-beads obtained were added to the amplification mix and oil, and vigorously shaken on a Tissue Lyser II (Qiagen) to create “micro-reactors” containing both amplification mix and a single bead. Emulsion was dispensed into a 96-well plate and the PCR amplification program was run according to the manufacturer’s recommendations. A 20 ng aliquot of each sample DNA was used for a 50 μL PCR reaction. The 16S universal primers 27F (5′ GAGTTTGATCMTGGCTCAG 3′) and 800R (5′ TACCAGGGTATCTAATCC 3′) were used for amplifying 16S rRNA genes (V1-V4 region, ~576–682 bp). The FastStart High Fidelity PCR System (Roche) was used for PCR under the following conditions: 94°C for 3 min; followed by 35 cycles of 94°C for 15 sec, 55°C for 45 sec, and 72°C for 1 min; and a final elongation step at 72°C for 8 min. After the PCR reaction, products were purified using AMPure beads (Beckman Coulter).

#### Next-generation sequencing

The sequencing run was performed by Macrogen Ltd. (Seoul, Republic of Korea). Following PCR amplification, the emulsion was chemically broken and the beads carrying the amplified DNA library were recovered and washed by filtration. Positive beads were purified using the biotinylated primer A (complementary to adaptor A), which binds to streptavidin-coated magnetic beads. The DNA library beads were next separated from the magnetic beads by melting the double-stranded amplification products, leaving a population of bead-bound single-stranded template DNA fragments. The sequencing primer was then annealed to the amplified single-stranded DNA. Lastly, beads carrying amplified single-stranded DNA were counted with a Particle Counter (Beckman Coulter). Sequencing was performed on a Genome Sequencer FLX plus (454 Life Sciences), and each sample was loaded in 1 region of a 70 mm × 75 mm PicoTiter plate (454 Life Sciences) fitted with an 8-lane gasket.

#### Selection of 16S rRNA and taxonomic assignment

All the sequence reads were compared to the Silva rRNA database using BLAST. Sequence reads that had a similarity score with an E-value < 0.01 were admitted as partial 16S rRNA sequences. Fewer than 1% of the sequence reads were considered non-16S rRNA sequences. CD-HIT-OTU software (http://weizhongli-lab.org/cd-hit-otu/) was used for clustering for identifying OTUs. The followings were removed for quality filtering: ambiguous bases; short reads (<188 bp); Chimera; homopolymer run. OTUs with cluster cut off of 97% sequence identity were clustered using UCLUST software. Quantitative Insight into Microbial Ecology (QIIME) software (package 1. 8. 0) was used for analyzing microbial communities including demultiplexing and quality filtering, OTU picking, taxonomic assignment, phylogenetic reconstruction, diversity analyses, and visualizations [15]. Taxonomic assignment of the sequence reads was performed using the NCBI Taxonomy and Nucleotide databases; the five most similar sequences to each sequence read were determined using BLAST bit scores and E-values. The Needleman-Wunsch global alignment algorithm was used to find the optimum alignment of two sequences along their entire length. A pairwise global alignment was performed on selected candidate hits to identify the best alignment. The taxonomy of the sequence with the highest similarity was assigned to the sequence read. Using similarity, isolates were identified as follows: species with > 97% similarity, genus > 94%, family > 90%, order > 85%, class > 80%, and phylum > 75%.

### Sample preparation and endogenous metabolite profiling

Extracts of the contents of the large intestine were prepared for metabolite profiling. The intestinal contents were extracted in 1 mL of 80% methanol by sonication for 10 min. After centrifugation (12,578 *g*, 4°C, 10 min), the supernatant was filtered using a 0.2-μm PTFE filter, and dried using a speed vacuum concentrator. The dried extracts were re-dissolved with 80% methanol (10mg/ml) for UPLC-Q-TOF-MS analysis under previously described analytical conditions [16]. For GC-TOF-MS analysis, dried samples were oximated with 50 μL of methoxyamine hydrochloride (20 mg/mL in pyridine) for 90 min at 30°C, and silylated with 50 μL of MSTFA for 30 min at 37°C. GC-TOF-MS analysis was performed using an Agilent 7890 gas chromatograph system (Agilent Technologies, Palo Alto, CA, USA), an Agilent 7693 auto-sampler (Agilent Technologies), and a Pegasus^®^ HT TOF MS (LECO, St. Joseph, MI, USA) system under previously described analytical conditions [13].

### Analysis of green tea catechin and exogenous metabolites

To identify the structure of green tea catechin exogenous metabolites, pooled large intestinal contents and liver tissue extracts were reconstituted in 100 μL of 80% methanol. Each sample aliquot (5 μL) was injected into the LC-triple-Q-MS system. The sample analysis was performed on a Nexera2 LC system (Shimadzu Corporation, Kyoto, Japan) connected to a triple quadrupole mass spectrometer (LC-MS 8040; Shimadzu) equipped with an electrospray ionization source in negative mode. The chromatographic separation was performed on a Kinetex C18 column (100 × 2.1 mm, 2.6 μm, Phenomenex, Torrance, CA). The column was eluted with solvent A (water containing 0.1% formic acid) and solvent B (acetonitrile containing 0.1% formic acid) at a flow rate of 200 μL/min. Gradient elution was conducted as follows: B was linearly increased form 5% to 70% over 7 min and then decreased to 5%. ESI source settings were as follows: capillary voltage -3000 V, vaporizer temperature 300°C, capillary temperature 350°C, sheath (Neb) gas 3 L/min, ion sweep gas 2.0 Arb, Aux gas 10 Arb, and drying gas 8 L/min. Previously reported catechin metabolites [17–19] were monitored, and the product ion scan mass spectra were obtained for each by MRM mode. The structures of catechin metabolites were identified by comparison with the MS/MS fragmentation patterns of commercially available catechin standards and our in house catechin library [20].

### Data processing and multivariate statistical analysis

The GC-TOF-MS data were acquired and preprocessed using the LECO Chroma TOF^™^ software (version 4.44; LECO Corp.) and converted into the NetCDF format (*.cdf) using the LECO Chroma TOF^™^ software. The UPLC-Q-TOF-MS data were acquired with MassLynx software (version 4.1; Waters Corp.), and raw data files were converted into NetCDF format (*.cdf) using MassLynx DataBridge (version 4.1; Waters Corp.). After conversion, the peak detection, retention time correction, and alignment were processed using the Metalign software package (http://www.metalign.nl). The resulting data were exported to an Excel file. Multivariate statistical analysis was conducted using SIMCA-P+ (version 12.0, Umetrics; Umea, Sweden). The data sets were auto-scaled (unit variance scaling) and mean-cantered in a column-wise fashion. Partial least squares-discriminant analysis (PLS-DA) was performed to compare each data set. The significance of PLS-DA model was determined by analysis of variance testing of cross-validated predictive residuals in SIMCA-P^+^ program. The variables were selected based on VIP value. The selected metabolites were putatively identified by comparing their retention time, mass spectra, and mass fragment patterns with those for commercial standard compounds analyzed under identical conditions and various commercially available databases, such as the National Institutes of Standards and Technology (NIST) library, Wiley 8, and the Human Metabolome Database (HMDB; http://www.hmdb.ca/). Significant differences were determined by analysis of variance (ANOVA) and Duncan’s multiple range tests using PASW Statistics 18 software (SPSS Inc.; Chicago, IL, USA). The linear discriminant analysis effect size (LEfSe) operation was performed using the online software (https://huttenhower.sph.harvard.edu/galaxy/). Pair-wise correlations between the intestinal microbiome and large intestinal endogenous metabolites were calculated using Pearson’s correlation coefficient test with average values of each groups, as implemented by PASW Statistics 18. The correlation network was constructed using endogenous metabolites and bacterial classes according to Pearson’s correlation coefficient (> ± 0.99) using the Cytoscape software (version 3.4.0).

## Results

### The green tea supplements (GTS) modulate large intestinal microbiome altered through UVB exposure in mice groups

**Fig 2:**
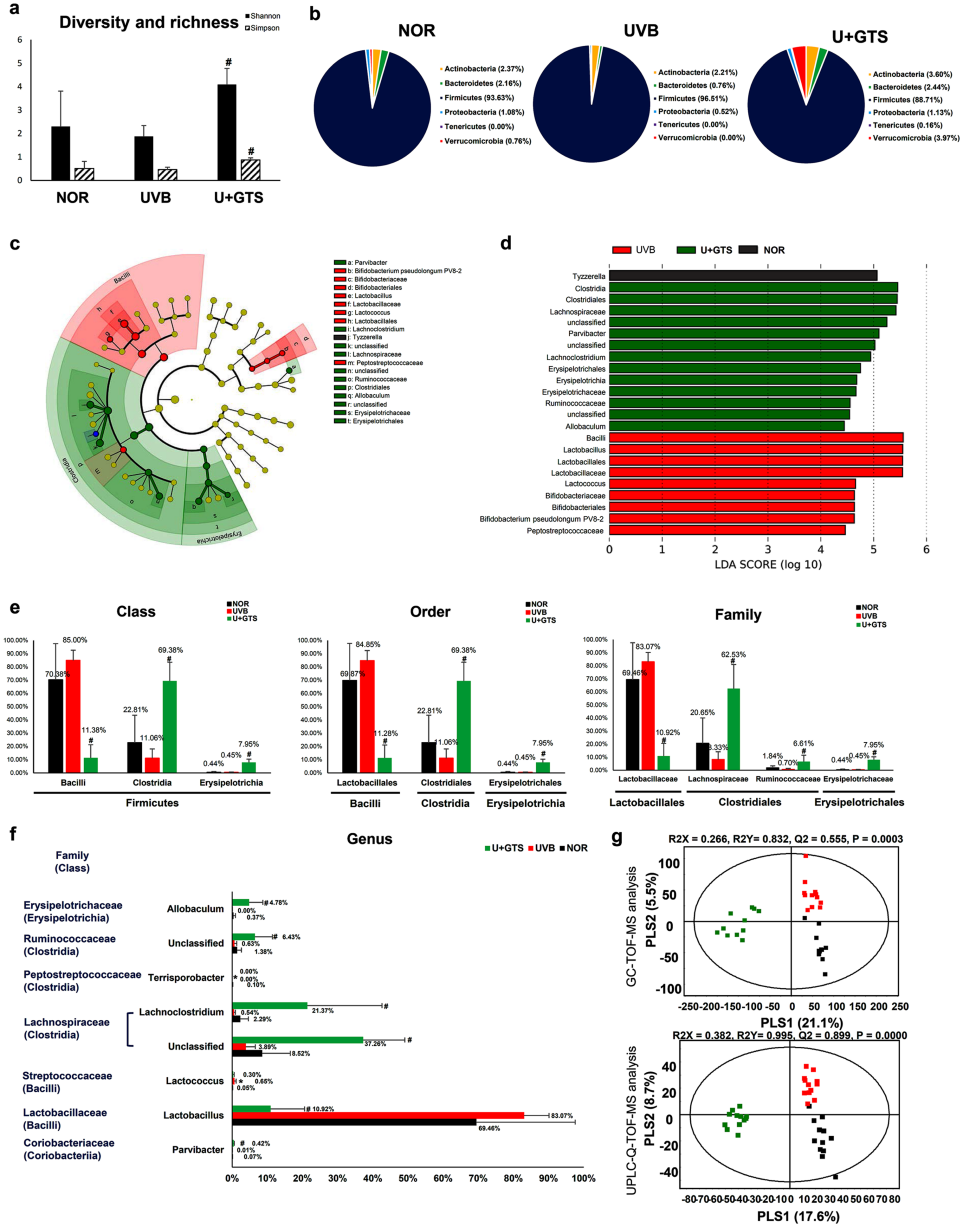
Comparison of the intestinal microbiome in the NOR, UVB, and U+GTS groups. a, Shannon and Simpson’s diversity indices; b, relative abundances of operational taxonomy units (OTUs) at the phylum level; c, histogram of the LDA scores of differentially abundant taxa as calculated by LEfSe; d, the LEfSe cladograms of six-level (from kingdom to genus) representing differentially abundant taxa; e, relative abundances of intestinal microbiome OTUs compared between groups in class, order, and family level; f, relative OTU abundance differentials between groups in genus level; g, partial least square-discriminated analysis score scatter plots of mouse intestinal content extracts analyzed by GC-TOF-MS and UPLC-Q-TOF-MS. The different letters indicate significant differences, as determined by ANOVA followed by Duncan’s multiple range tests (*p* < 0.05). Black filled square-NOR group; red filled square-UVB group; green filled square- U+GTS group.

We investigated the *in vivo* mitigation of the detrimental effects of chronic UVB exposure through green supplements (GTS) on the large intestinal microbial communities and exo/endogenous metabolomes using mice model. The Shannon and Simpson’s diversity indices for gut microbiomes showed that the overall microbial assortments in UVB groups were reduced slightly but notably compared to the NOR group. On the other hand, the U+GTS group exhibited an enhanced microbiome diversity compared to other test groups (Fig 2a). Fig 2b shows inter-group differences for the relative abundances of bacterial operational taxonomic units (OTUs) at the phylum level (*Actinobacteria*, *Bacteroidetes*, *Firmicutes*, *Proteobacteria*, *Tenericutes*, and *Verrucomicrobia*). The proportion of the phyla *Bacteroidetes*, *Proteobacteria*, and *Verrucomicrobia* were decreased while those of *Firmicutes* was increased in the UVB group relative to the NOR group. Intriguingly, in U+GTS group, the proportion of all phyla, except *Firmicutes*, was higher than the other two groups. To determine major abundance patterns of bacterial taxa in experimental groups, we processed microbial community analysis data using linear discriminant analysis effect size (LEfSe) analytical method [21] (Fig 2c and 2d). The LEfSe circular cladogram, with six-levels ranging from kingdom to genus, highlighted the class *Bacilli* and order *Bifidobacteriales* as the most discriminant bacterial taxa among UVB-exposed mice group, while the classes *Clostridia* and *Erysipelotrichia* were highlighted as the most discriminant bacterial taxa in U+GTS group. In addition, to compare the relative abundances of intestinal microbial taxa in response to UVB exposure with GTS, significantly modulated bacteria were selected based on relative OTU abundance. A total of 3 in 14 classes (*Bacilli*, *Clostridia*, and *Erysipelotrichia*), 3 in 16 orders (*Lactobacillales*, *Clostridiales*, and *Erysipelotrichales*), 3 in 29 families (*Lactobacillaceae*, *Lachnospiraceae*, *Erysipelotrichaceae*), and 7 in 46 genera differed significantly among the test mice groups (Fig 2e and 2f). At the genus level, 7 out of 46 genera showed differences among the mice groups with UVB triggered an increase in *Lactobacillus* and *Lactococcus*, whereas GTS induced the enrichment of *Allobaculum*, *Parvibacter*, *Lachnoclostridium*, and two unclassified taxa from *Ruminococcaceae* and *Lachnospiraceae* families.

### Correlations for intestinal endogenous metabolomes and microbiomes effected through GT supplementation in mice groups subjected to UVB exposure

The mass spectrometry (MS) based endogenous metabolite profiling from the large intestinal contents was performed to evaluate the effects of UVB exposure and GTS treatment in mice groups. The PLS-DA score plots revealed that all the three groups were distinct from each other with statistically significant model values *viz*., R^2^X_(cum)_, R^2^Y_(cum)_, and Q^2^_(cum)_, indicating its fitness and prediction accuracy at *p*-value (<0.05) obtained through cross-validation (Fig 2g). A total of 58 significantly discriminant endogenous metabolites were putatively identified among the intestinal contents of NOR, UVB, and U+GTS mice groups, based on the variable importance in projection (VIP) value (> 0.7) and *p*-value (< 0.05). These discriminant metabolites included 13 amino acids, 4 organic compounds, 3 nucleobases, 9 carbohydrates, 5 fatty acids, 18 lipids, and 6 bile acids. Detailed information and fold change ratios converted from relative metabolite levels are presented in Table 1. Based on the ratios of average metabolite levels, lysophospholipids were remarkably decreased in the UVB group compared to the NOR group. Relative levels of most amino acids, fatty acids, lipids, and bile acids were lower in the U+GTS group than the NOR group, while metabolite levels of nucleobases and carbohydrates were higher in the U+GTS group. The following metabolites showed particularly high variation: amino acids—asparagine (**1**), tryptophan (**11**), and valine (**13**); organic compounds—malic acid (**14**) and succinic acid (**16**); nucleobase—uracil (**20**); carbohydrates—fructose (**22**), glyceric acid (**24**), and mannose (**26**); fatty acids—linoleic acid (**31**), oleic acid (**32**), and palmitoleic acid (**34**); lipids—lanosterol (**35**), monoolein (**36**), monopalmitin (**37**), monoacylglycerol (18:4) (**38**), lysoPE (15:0) (**39**), lysoPC (18:0) (**45**), lysoPE (18:0) (**47**), lysoPE (18:1) (**49**), lysoPE (18:2) (**50**), lysoPC (20:1) (**51**), and lysoPC (20:1) (**52**); the bile acids- taurocholic acid (**53**; **55**), taurodeoxycholic acid (**54**), and deoxycholic acid (**57**).

**Table 1:** The major endogenous metabolites in the large intestinal contents altered by chronic UVB irradiation and GTS, as analyzed by GC-TOF-MS and UPLC-Q-TOF-MS.

No.	Tentative Metabolites^a^	Ratio of average	No.	Tentative Metabolites^a^	Ratio of average
		(NOR/UVB/U+GTS)			(NOR/UVB/U+GTS)
***Amino acids***			***Fatty acids***		
**1**	Asparagine	1.00 / 0.81 / **0.14**	**30**	Hexanoic acid	1.00 / 0.71 / 0.54
**2**	Isoleucine	1.00 / 0.98 / 0.74	**31**	Linoleic acid	1.00 / 1.01 / **0.36**
**3**	Leucine	1.00 / 0.92 / 0.51	**32**	Oleic acid	1.00 / 1.01 / **0.26**
**4**	Lysine	1.00 / 0.90 / 0.55	**33**	Palmitic acid	1.00 / 1.18 / 0.66
**5**	Methionine	1.00 / 0.98 / 1.56	**34**	Palmitoleic acid	1.00 / 0.87 / **0.15**
**6**	Ornithine	1.00 / 0.75 / 0.77	***Lipids***		
**7**	Phenylalanine	1.00 / 1.01 / 0.90	**35**	Lanosterol	1.00 / 1.05 / **0.41**
**8**	Proline	1.00 / 1.01 / 0.82	**36**	Monoolein	1.00 / 1.07 / **0.12**
**9**	Serine	1.00 / 1.00 / 0.70	**37**	Monopalmitin	1.00 / 0.85 / **0.03**
**10**	Threonine	1.00 / 1.02 / 0.77	**38**	Monoacylglycerol (18:4)	1.00 / 1.74 / **0.46**
**11**	Tryptophan	1.00 / 0.85 / **0.28**	**39**	LysoPE(15:0)	1.00 / 1.38 / **8.82**
**12**	Tyrosine	1.00 / 0.97 / 0.83	**40**	LysoPC(16:0)	1.00 / 0.73 / 0.89
**13**	Valine	1.00 / 0.92 / **0.40**	**41**	LysoPC(16:0)	1.00 / 0.75 / 1.12
***Organic compounds***			**42**	LysoPE(16:0)	1.00 / 0.66 / 0.79
**14**	Malic acid	1.00 / 1.38 / **23.6**	**43**	LysoPE(16:0)	1.00 / 0.66 / 0.88
**15**	Pyruvic acid	1.00 / 1.12 / **0.30**	**44**	LysoPE(dm16:0e)	1.00 / 0.58 / 1.27
**16**	Succinic acid	1.00 / 1.23 / **7.05**	**45**	LysoPC(18:0)	1.00 / 0.64 / **0.48**
**17**	*trans*-Urocanic acid	1.00 / 1.33 / 0.64	**46**	LysoPC(18:0)	1.00 / 0.72 / 0.64
***Nucleobases***			**47**	LysoPE(18:0)	1.00 / 0.70 / **0.32**
**18**	Hypoxanthine	1.00 / 1.09 / 1.31	**48**	LysoPC(18:1)	1.00 / 0.75 / 0.72
**19**	Inosine	1.00 / **0.39** / 1.50	**49**	LysoPE(18:1)	1.00 / 0.67 / **0.50**
**20**	Uracil	1.00 / 1.22 / **2.13**	**50**	LysoPE(18:2)	1.00 / 0.94 / **0.49**
***Carbohydrates***			**51**	LysoPC(20:1)	1.00 / **0.30** / **0.16**
**21**	Fructose	1.00 / 1.32 / 1.99	**52**	LysoPC(20:1)	1.00 / 0.68 / **0.26**
**22**	Fructose	1.00 / 1.31 / **2.22**	***Bile acids***		
**23**	Fucose	1.00 / 1.16 / 0.78	**53**	Taurocholic acid^b^	1.00 / 1.05 / **0.13**
**24**	Glyceric acid	1.00 / 1.24 / **2.87**	**54**	Taurodeoxycholic acid^c^	1.00 / 1.44 / **0.07**
**25**	Glycerol	1.00 / 1.02 / 1.25	**55**	Taurocholic acid^b^	1.00 / 0.76 / **0.11**
**26**	Mannose	1.00 / 0.71 / **4.45**	**56**	Cholic acid^d^	1.00 / 1.14 / 1.77
**27**	myo-Inositol	1.00 / 0.99 / 1.06	**57**	Deoxycholic acid^e^	1.00 / 1.22 / **0.46**
**28**	Sucrose	1.00 / 1.01 / 3.17	**58**	Deoxycholic acid	1.00 / 1.13 / 0.66
**29**	Xylose	1.00 / 1.04 / 1.92			

Metabolites selected by VIP > 0.7 from PLS-DA model (Fig 2g) and *p* < 0.05 of one-way ANOVA analysis. Relative contents rations were normalized by NOR group. LysoPC, lysophosphatidylcholine; LysoPE, lysophosphatidylethanolamine LysoPC and lysoPE were detected as two different forms which contain the fatty acyl chain at sn-1 and sn-2 site on the backbone of glycerol.

^a^ Assignment of metabolites was carried out using HMDB, NIST, Wiley 8, high resolution mass data (ppm), and in house library

^b^ It identified as taurine conjugated with cholic acid affiliation (taurochlic acid or tauro-muricholic acid or tauroallocholic acid or taurohyocholic acid, tauroursocholic acid)

^c^ It identified as taurine conjugated with deoxycholic acid affiliation (taurodeoxycholic acid or tauroursodeoxycholic acid or taurochenodesoxycholic acid)

^d^ It identified as cholic acid affiliation (cholic acid or muricholic acid or lithocholic acid)

^e^ It identified as deoxycholic acid affiliation (deoxycholic acid or ursodeoxycholic acid or chenodeoxycholic acid)

Bold values indicate the fold change > 2.0 or < 0.5 compared to NOR group

**Fig 3:**
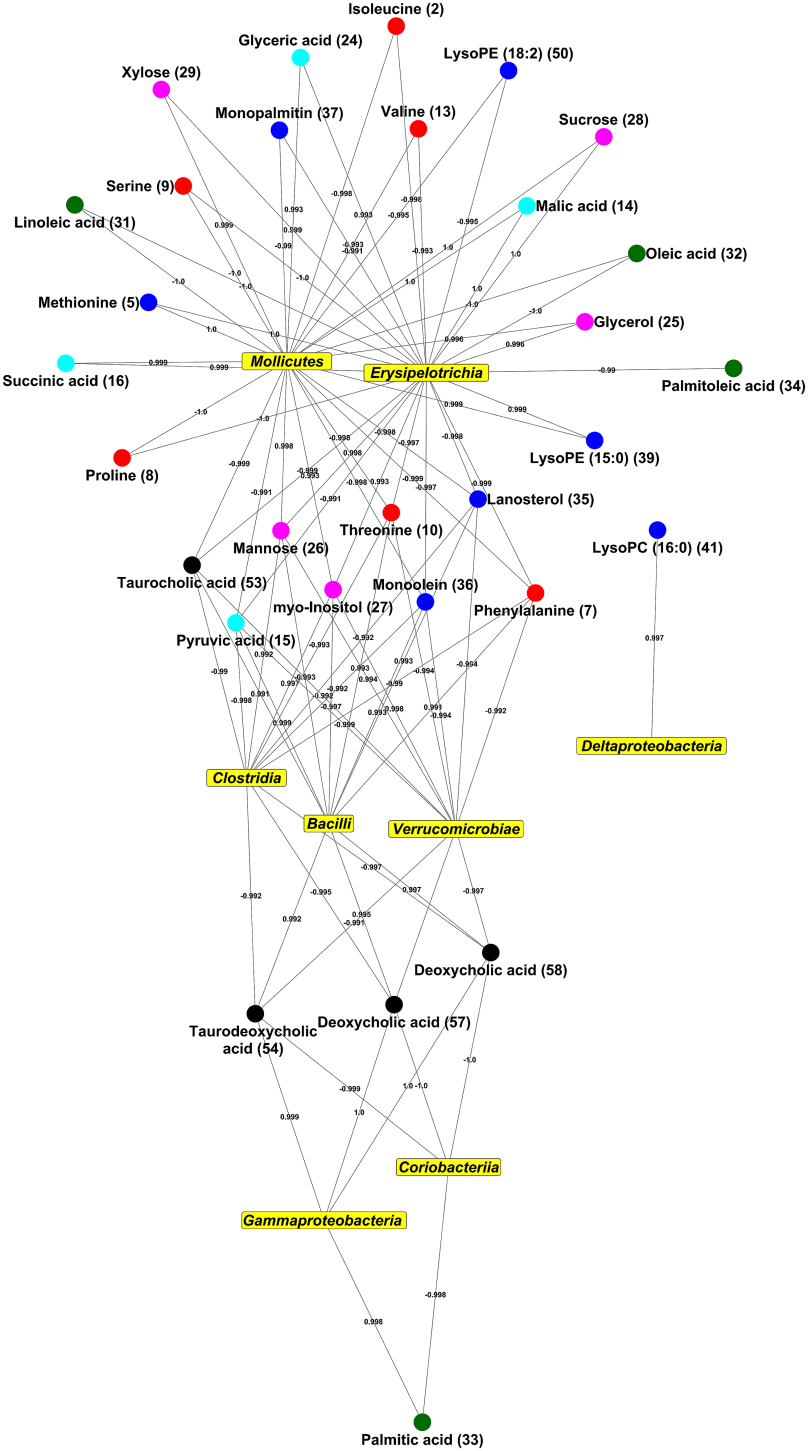
orrelation network of intestinal microbiome and endogenous metabolites of large intestinal contents according to pearson’s correlation coefficient (-0.99<r<0.99). C Numbers on metabolites were identical on Table 1.

The correlation analysis between the large intestinal microbiome and endogenous metabolites showed that the bacteria in the classes *Bacilli*, *Clostridia*, and *Erysipelotrichia*, which were significantly discriminated between experimental mice groups, showed high positive and negative correlations with intestinal endogenous metabolites (Fig 3). Especially, *Clostridia* and *Bacilli* showed high correlations with bile acids including deoxycholic acid, taurodeoxycholic acid, and taurocholic acid.

### The exogenous metabolic derivatives of green tea catechin in large intestinal contents and liver sample extracts from UVB irradiated mouse with/without GTS

**Fig 4:**
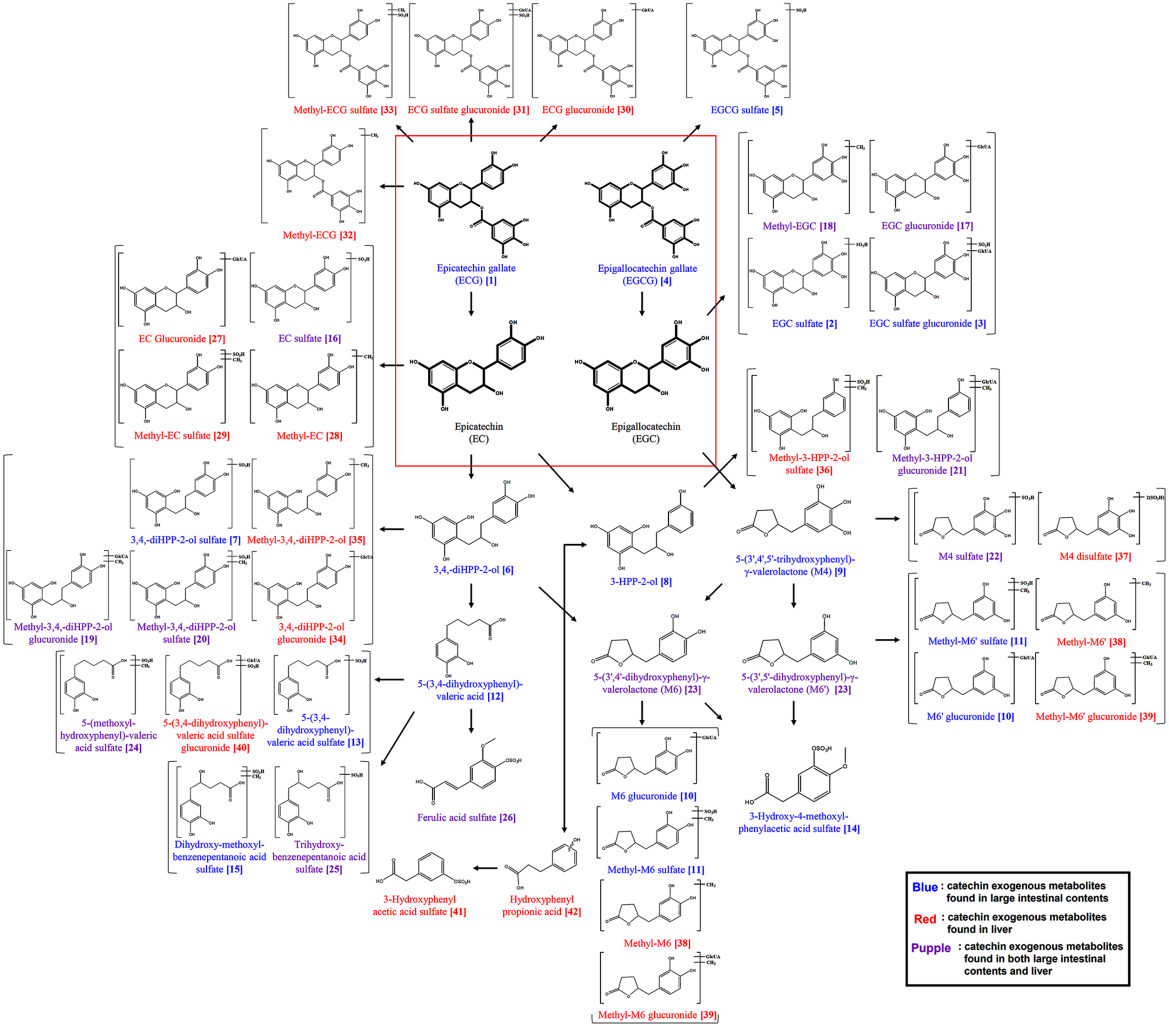
Proposed catechin exogenous metabolism pathway using preliminarily identified various transformed green tea catechins in the large intestine and the liver. Blue text indicates exogenous catechin metabolites found in the large intestinal contents; Red text indicates exogenous catechin metabolites found in the liver; Purple text indicates exogenous catechin metabolites found in both the large intestinal contents and the liver. Numbers on metabolites were identical on Table 2.

A total of 26 and 27 catechin metabolites were detected from the large intestinal contents and liver samples, respectively (Table 2 and Fig 4). The gallated catechins including free form ECG and EGCG were observed in the large intestinal contents. Alternatively, the non-gallated catechins including EC and EGC were detected as conjugated derivatives from the large intestine contents as well as liver samples. A variety of diverse methylated catechin metabolites were also found associated with liver samples. In addition, free form and derivatives of ring fission products and phenolic acids related to green tea catechins were also detected variously from the large intestinal contents and liver samples from U+GTS mice groups. A number of exogenous metabolites detected include the ring fission products *viz*., derivatives of 1-(3',4'-dihydroxyphenyl)-3-(2'',4'',6''-trihydroxyphenyl)-propan-2-ol (3,4-diHPP-2-ol), 3-HPP-2-ol, 5-(3',4',5-trihydroxyphenyl)-γ-valerolactone (M4), 5-(3'4'-dihyroxyphenyl)-γ-valerolactone/5-(3'5'-dihydroxyphenyl)-γ-valerolactone (M6/M6'), and 5-(3,4,-Dihydroxyphenyl)-valeric acid. Additionally, some phenolic acids including the derivatives of benzenepentanoic acid, phenylacetic acid, phenylpropionic acid, and ferulic acid were also observed (Table 2 and Fig 4).

**Table 2:** Preliminary identification of exogenous metabolites of green tea catechins in the large intestinal contents and the liver.

No.	Compounds*	Location	[M-H]^-^ (*m/z*)	Fragment ion (m/z)
1	ECG	Large intestine	441	169
2	EGC sulphate	Large intestine	385	305
3	EGC sulphate glucuronide	Large intestine	561	305
4	EGCG	Large intestine	457	169
5	EGCG sulphate	Large intestine	537	457
6	3,4-diHPP-2-ol	Large intestine	291	247
7	3,4-diHPP-2-ol sulphate	Large intestine	371	291
8	3-HPP-2-ol	Large intestine	275	231
9	M4	Large intestine	223	123/179
10	M6/M6' glucuronide	Large intestine	383	207
11	Methyl M6/M6' sulphate	Large intestine	301	221
12	5-(3,4,-Dihydroxyphenyl)-valeric acid	Large intestine	209	106/101
13	5-(3,4,-Dihydroxyphenyl)-valeric acid sulphate	Large intestine	289	209
14	3-Hydroxy-4-methoxyl-phenylacetic acid sulphate	Large intestine	261	122
15	Dihydroxy-methoxyl-benzenepentanoic acid sulphate	Large intestine	319	162/239/221/177
16	EC sulphate	Large intestine, Liver	369	289/245/207/189/181/163
17	EGC glucuronide	Large intestine, Liver	481	305/261/175/137
18	Methyl EGC	Large intestine, Liver	319	275/257/245/195/167/137/109
19	Methyl 3,4-diHPP-2-ol glucuronide	Large intestine, Liver	481	305/175/149/135/85
20	Methyl 3,4-diHPP-2-ol sulphate	Large intestine, Liver	385	305/149
21	Methyl 3-HPP-2-ol glucuronide	Large intestine, Liver	465	151/135/125
22	M4 sulphate	Large intestine, Liver	303	151/135/108/92
23	M6/M6'	Large intestine, Liver	207	135/85/73/59
24	5-(Methoxyl-hydroxyphenyl)-valeric acid sulphate	Large intestine, Liver	303	151/135/109/92
25	Trihydroxybenzene pentanoic acid sulphate	Large intestine, Liver	305	164/135/123
26	Ferulic acid sulphate	Large intestine, Liver	273	193/175/149/134/106
27	EC glucuronide	Liver	465	203/135
28	Methyl EC	Liver	303	245/151/135
29	Methyl EC sulphate	Liver	383	245/203/179/161/159/125
30	ECG glucuronide	Liver	617	289/245
31	ECG sulphate glucuronide	Liver	697	135
32	Methyl ECG	Liver	464	289/219/193
33	Methyl ECG sulphate	Liver	535	135
34	3,4-diHPP-2-ol glucuronide	Liver	467	291/207/175/167/135/125
35	Methyl 3,4-diHPP-2-ol	Liver	305	149/135/125/108
36	Methyl 3-HPP-2-ol sulphate	Liver	369	217/173/151/125
37	M4 disulphates	Liver	383	303/221/203
38	Methyl M6/M6'	Liver	221	163/147/85/73
39	Methyl M6/M6' glucuronide	Liver	397	163/147/73
40	5-(3,4-Dihydroxyphenyl)-valeric acid sulphate glucuronide	Liver	165	135
41	3-Hydroxyphenylacetic acid sulphate	Liver	231	151/107/93
42	Hydroxyphenyl propionic acid	Liver	165	121/77

* ECG, epicatechin gallate; EGC, epigallocatechin; EGCG, epigallocatechin gallate; 3,4-diHPP-2-ol, 1-(3',4'-dihydroxyphenyl)-3-(2'',4'',6''-trihydroxyphenyl)-propan-2-ol; 3-HPP-2-ol, 1-(3'-hydoxyphenyl)-3-(2'',4'',6''-trihydroxyphenyl)-propan-2-ol; M4, 5-(3',4',5-trihydroxyphenyl)-γ-valerolactone; M6, 5-(3'4'-dihyroxyphenyl)-γ-valerolactone; M6', 5-(3'5'-dihydroxyphenyl)-γ-valerolactone; EC, epicatechin

## Discussion

In this study, we investigated the effects of GTS on the large intestinal microbiome and exo/endogenous metabolomes for the mice groups subjected to the gradients of chronic UVB-doses using integrated microbiome-metabolome analyses. In the intestine, the metabolome is generated by diverse organisms under particular environmental conditions, and studying it can provide insight into the dynamic operations and functional contributions of microbial communities [22]. We observed that UVB irradiation caused mild but notable changes in the large intestinal microbiome, as shown with an increased proportion of *Firmicutes*. In particular, classes *Bacilli* and *Bifidobacteriales* were the discriminant bacterial taxa for UVB group (Fig 2). In general, the gut microbial communities are reportedly sensitive to various external perturbations *viz*., genetics, age, environment, and diet [23]. However, the experimental reports substantiating the correlations among the altered intestinal microbiomes and skin health pertaining to the chronic UVB irradiation, as a primary environmental perturbation, are lacking. Alternatively, there are reports that UV irradiation induce immunomodulation in intestine and inhibits intestinal tumor growth [24, 25]. Hence, one can presume a high possibility towards the influence of protracted UV irradiation on intestinal micro-environments, an aspect relatively unexplored. In our study, we observed a slight but notable disparity in intestinal microbial communities for UVB group compared to the NOR group, which we conjectured for the resilient nature of gut microbiomes. However, the prolonged UV irradiation reportedly causes inflammatory response leading to the acute disturbances of intestinal microbiomes [26]. Intriguingly, the diet, one of the crucial factors in shaping intestinal microbiome can potentially considered a mean towards revitalizing the disturbed microbiome [23]. In our study, we maintained a normal diet for both the NOR and the UVB mice groups for entire experimental period, which might be the reason for mild differences in the microbiome of two groups. However, as shown in Fig 2f, genus *Lactobacillus* and *Lactococcus*, showed increasing patterns in UVB group as compared to NOR group. The genus *Lactobacillus* is probiotic and is also known to stimulate the immune system through regulating the intestinal microbiome colonization [27, 28]. As reported earlier, the oral administration of *bifidobacterium* and *Lactococcus* species specifically induce the UV photo-protective effects and promote skin health, respectively [7, 29–30]. Hence, we can assume a parallel mechanism behind the rise in probiotic bacterial genera following the UVB treatment of mice groups. Additionally, the observed congruency with published literatures suggests a potential correlation between the chronic UVB irradiation and the overall modulation of the intestinal microbiomes.

In UVB-exposed mice with GTS administration (U+GTS), a significantly enriched microbial community diversity was observed, especially, with higher abundance of *Allobaculum*, *Lachnoclostridium*, *Parvibacter*, and unclassified taxa from *Ruminococcaceae* and *Lachnospiraceae* (Fig 2c), as compared to UVB and NOR groups. These high differences might be credited to the competition among the members of the large intestinal microbiome towards the limiting green tea metabolites or its bio-transformed catabolite products. In general, the green tea catechins are actively transformed into various catabolite products by gut microbiota. In this study, various derivatives of four green tea catechins including EC, ECG, EGC, and EGCG along with their exogenous metabolites *i*.*e*., ring fission products and phenolic acids, were primarily detected from the large intestinal contents and liver sample extracts (Table 2 and Fig 4). These exogenous metabolites potentially play two different roles: (a) the modulation of the gut microbiome, and (b) act as bioactive compounds in body [31, 32]. In a conspicuous way, free forms of gallated catechins (ECG and EGCG) were also observed from the large intestinal contents, whereas, the conjugated derivatives of non-gallated catechins (EC and EGC) were additionally detected from the liver samples of the treated (U+GTS) mice groups. Diverse methylated exogenous metabolites of catechin were found in the liver (Fig 4, Table 2). These results are consistent with a previous report that non-gallated catechins and their methylated forms were more bioavailable than gallated catechins [33]. Among the total 42 exogenous metabolites, several are reported to possess antibacterial, antioxidant, and anti-inflammatory effects. The major phenolic acids formed during microbial fermentation of green tea catechins, including benzoic acid, phenylacetic acid, and phenylpropionic acids, inhibit the growth of pathogenic bacteria including *Clostridium* spp., *Bacteroides* spp., *Escherichia coli*, *Staphylococcus aureus*, *Pseudomonas aeruginosa*, and *Candida albicans* [34, 35]. The exogenous catechin metabolites in liver might be linked to the beneficial health effects and associated bioactivities of green tea. The EC and its methylated metabolites have antioxidant activity [36]. In addition, phenolic acids, such as 3-phenylpropionic acid, 3-hydroxyphenylacetic acid, and 3-(4-hydroxyphenyl)-propionic acid, are known to decrease the cyclooxygenase-2 (COX-2) protein level and to induce stimuli such as growth factors, cytokines, TNF-α, and lipopolysaccharides [37]. Hence, we propose that *in vivo* transformed catechin derivatives and phenolic acids potentially affect the gut micro-environment in the large intestine as well as in body, which together modulates the gut microbiome leading to antioxidant and anti-inflammatory effects.

Furthermore, we observed a clear variation in the endogenous metabolite levels from large intestinal contents following the chronic UVB irradiation and/or GTS dietary administration in mice groups. A significant decrease in lysophospholipids levels were observed in the UVB groups compared to the NOR group. On the other hand, the GTS induced the significant alteration in large intestinal endogenous metabolomes including amino acids, organic compounds, nucleobases, carbohydrate, fatty acids, lipids, and bile acids (Table 1). The green tea reportedly influences intestinal absorption of various nutrients *viz*., dietary fats, cholesterol, lipids, and glucose, which might be correlated with the observed alterations in endogenous metabolite levels from large intestinal contents [38, 39]. Further, the altered endogenous metabolite levels showed the high positive and negative correlations with intestinal microbiomes suggesting their pivotal roles in gut microbial metabolism linked to carbohydrate, lipid, and bile acids (Fig 3). These metabolites are also known to have close relation with carbohydrate metabolism effecting complex interactions between gut microbial and host enzymatic reactions [40]. We observed that the relative levels of monosaccharides *viz*., fructose, mannose, and xylose from the large intestinal contents were significantly increased in the U+GTS group. In addition, the GTS resulted in a significant decrease in taurine conjugated bile acids and secondary bile acids (deoxycholic acid) abundance, coupled with an increase in primary bile acid *i*.*e*., cholic acid levels (Table 1). When bile acid absorption occurs in the liver, co-absorption of nutrients, including lipids and glucose occurs, which are related to hepatic TG, glucose, and energy homeostasis, occurs simultaneously [41]. However, EGCG has been reported to inhibit the ileal apical sodium dependent bile acid transporter activity [42]. In this study, the GTS might have influenced the bile acid absorption, resulting in significant reduction in the levels of bile acids from large intestinal contents. Additionally, the bile acids might have bio-transformed supposedly through 7-αβ-dehydroxylation reactions effected by gut bacterial genera *viz*., *Clostridium*, *Bacteroides*, *Lactobacillus*, *Bifidobacteria*, and *Enterobacter* [43, 44]. The high levels of antimicrobial primary bile acids, such as cholic acid, also influence the gut microbiome composition, particularly the abundance of *Firmicutes* [45]. As per the correlation network analysis, various secondary bile acids showed high correlations with bacterial classes such as *Clostridia*, *Bacilli*, and *Verrucomicrobiae*. The UVB irradiation with GTS dietary administrations most significantly reduced the *Firmicutes* abundance including *Clostridia* and *Bacilli*, which might be correlated to the reduction in the levels of bile acids in large intestinal contents (Fig 2 and Table 1).

In conclusion, we outlined an *in vivo* integrated microbiome-metabolome methodology towards probing the GTS mediated modulation of large intestinal microbiome and metabolome altered through UVB exposures. The GTS revamped the microbial community diversity through maneuvering the altered states of various endogenous metabolites in mice groups subjected to UVB-exposure. Hence, we propose that the overall changes in exo/endogenous metabolites levels influenced by GTS administration could be have enriched the gut microbiome, and thus can potentially mitigate the deleterious effects of chronic UVB irradiation.

## Supporting information

S1 Checklist The ARRIVE guidelines checklist. (PDF)
